# Neutrophil percentage‐to‐albumin ratio and monocyte‐to‐lymphocyte ratio as predictors of free‐wall rupture in patients with acute myocardial infarction

**DOI:** 10.1002/jcla.24136

**Published:** 2021-11-25

**Authors:** Kai Dai, Zhibing Li, Yafei Luo, Qianhui Xiong, Yao Xiong, Zhifang Song, Wenjun Xiong

**Affiliations:** ^1^ Department of Cardiovascular Medicine The First Affiliated Hospital of Nanchang University Medical Department of Nanchang University, Nanchang Jiangxi China; ^2^ Department of Intensive Care Unit The First Affiliated Hospital of Nanchang University Medical Department of Nanchang University, Nanchang Jiangxi China; ^3^ Department of Cardiovascular Medicine Jiangxi Provincial People’s Hospital Affiliated to Nanchang University Medical Department of Nanchang University, Nanchang Jiangxi China

**Keywords:** acute myocardial infarction, free‐wall rupture, inflammatory, monocyte‐to‐lymphocyte ratio, neutrophil percentage‐to‐albumin ratio

## Abstract

**Backgrounds:**

Free‐wall rupture (FWR) has a high mortality rate. We aimed to find sensitive predictive indicators to identify high‐risk FWR patients by exploring the predictive values of neutrophil percentage‐to‐albumin ratio (NPAR) and monocyte‐to‐lymphocyte ratio (MLR) on patients with acute myocardial infarction (AMI).

**Methods:**

76 FWR patients with AMI were collected, and then 228 non‐CR patients with AMI were randomly selected (1:3 ratio) in this retrospective study. The independent influencing factors of FWR were evaluated by univariate and multivariate logistic regression analysis. The receiver‐operating characteristic (ROC) curve analysis was applied to evaluate the predictive value of NPAR and MLR for FWR.

**Results:**

According to the results of multivariate logistic regression analysis, emergency percutaneous coronary intervention (PCI) (OR = 0.27, 95% CI: 0.094–0.751, *p* = 0.012), angiotensin‐converting enzyme inhibitor (ACEI)/angiotensin receptor blocker (ARB) treatment (OR = 0.17, 95% CI: 0.044–0.659, *p* = 0.010), NPAR (OR = 2.69, 95% CI: 1.031–7.044, *p* = 0.043), and MLR (OR = 5.99, 95% CI: 2.09–17.168, *p* = 0.001) were the influencing factors of the FWR patients with AMI, independently. Additionally, the NPAR and MLR were the predictors of FWR patients, with AUC of 0.811 and 0.778, respectively (both *p *< 0.001).

**Conclusions:**

In summary, the emergency PCI and ACEI/ARB treatment were independent protective factors for FWR patients with AMI, while the increase of MLR and NPAR were independent risk factors. What's more, NPAR and MLR are good indicators for predicting FWR.

## Graphical abstract

Emergency PCI and ACEI/ARB treatment were independent protective factors for for FWR patients with AMI, while the increase of MLR and NPAR were independent risk factors. What's more, NPAR and MLR are good indicators for predicting FWR. This study provides evidence for a better understanding between NPAR, MLR and FWR.

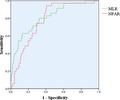

## 1 INTRODUCTION

Acute myocardial infarction (AMI) is the main type of acute coronary syndrome (ACS), and cardiac rupture (CR) is one of the most serious complications of AMI.^1^ According to the location, CR can be divided into ventricular free‐wall rupture (FWR), ventricular septal perforation (VSR), and papillary muscle rupture (PMR), among which FWR is the most common and dangerous. In recent years, with the popularization of reperfusion therapy, the incidence of CR after AMI was 1%‐3% and the mortality rate was above 75%.^2^, ^3^ Moreover, FWR had a higher mortality rate among CR due to the sudden onset, rapid progression and difficulty in treatment.^4^ Therefore, how to identify high‐risk FWR patients early and take active intervention measures are helpful to prevent and reduce the occurrence of FWR.

As novel inflammatory indicators, neutrophil percentage‐to‐albumin ratio (NPAR) and monocyte‐to‐lymphocyte ratio (MLR) are extracted from the hemogram. Among inflammation‐related diseases, the novel inflammatory indicators from the hemogram could predict the development and control levels of type 2 diabetes mellitus.^5^, ^6^, ^7^ MLR was also associated with diabetic nephropathy.^8^ Neutrophil‐to‐lymphocyte ratio, as a novel inflammatory indicator, may be useful to prove the presence of Hashimoto's thyroiditis and distinguish between malignant and benign thyroid nodules.^9^, ^10^ Besides, in patients with ulcerative colitis, neutrophil‐to‐lymphocyte ratio was associated with active disease.^11^ Moreover, previous study showed that with the rise of NPAR, the hospital mortality rate of AMI patients will also increase.^12^ Wang found that MLR was an independent predictor of major adverse cardiovascular events in patients with ST‐elevation myocardial infarction (STEMI).^13^ Therefore, we suspected that NPAR and MLR were related to AMI patients with FWR. In this study, the predictive values of NPAR and MLR on this population were discussed, so as to find sensitive predictive indicators to identify high‐risk patients with FWR as soon as possible.

## 2 MATERIALS AND METHODS

### 2.1 Study patients and design

A retrospective collection of patients with AMI diagnosed and admitted in the First Affiliated Hospital of Nanchang University and Jiangxi Provincial People's Hospital Affiliated to Nanchang University from January 2013 to September 2020.

The inclusion criteria were (1) meeting the diagnostic criteria for AMI according to the definition of ACC/AHA^14^; (2) fitting the diagnosis of FWR: the appearance of symptoms and signs of pericardial tamponade, electromechanical separation, and a large amount of pericardial effusion and pericardial puncture with noncoagulable fluid.^15^ The diagnosis of AMI and FWR were decided by two trained cardiologists. The exclusion criteria were (1) patients with other types of CR except FWR; (2) diagnosed with severe infection, liver and kidney disease, malignant tumors, and hematological diseases; (3) without complete clinical data. After determining FWR patients, non‐CR patients with AMI were randomly selected (1:3 ratio) in this retrospective study. As a result, a total of 304 eligible patients were included (FWR group: *n* = 76; non‐CR group: *n* = 228).

### 2.2 Data collection

General and hospitalization data of patients enrolled were collected. General information included age, gender, previous hypertension, diabetes mellitus, coronary artery disease (CAD), stroke, smoker, and drinker. The collection of hospitalization information included the level of systolic blood pressure (SBP), diastolic blood pressure (DBP), heart rate, blood cells, and biochemical indicators, especially NPAR and MLR at the first time after admission. Furthermore, the treatment after admission was analyzed, including the oral beta blockers, angiotensin‐converting enzyme inhibitor (ACEI)/angiotensin receptor blocker (ARB) and statin treatment, particularly reperfusion therapy through percutaneous coronary intervention (PCI).

### 2.3 Ethics statement

This research was an anonymous retrospective study, which was approved by the ethics committee of the First Affiliated Hospital of Nanchang University and Jiangxi Provincial People's Hospital Affiliated to Nanchang University. In accordance with national legislative and institutional requirements, participation in this study did not require written informed consent.

### 2.4 Statistical analysis

SPSS25.0 software (version 25.0 for Windows, SPSS, Inc, Chicago, Illinois) was used to analyze the data of continuous variables and dichotomous variables. The continuous variables conforming to normal distribution were expressed by mean ± standard deviation, and T‐test was applied to the comparison between groups. Otherwise, they were presented as median (interquartile range), and the Mann‐Whitney *U* test was used to compare the difference of the two groups. As for dichotomous variables, they were described by frequency (percentage), and χ² test was adopted. The potential risk factors of FWR were evaluated by univariate logistic regression analysis, and the indicators with *p *< 0.05 were screened out and introduced into multivariate logistic regression analysis, in order to find the independent risk factors of FWR. *P *< 0.05 was considered to be statistically significant. According to the receiver‐operating characteristic (ROC) curve analysis, the predictive values of NPAR and MLR for FWR were determined, and the result of area under the curve (AUC) >0.70 was regarded as a good predictive value.

## 3 RESULTS

### 3.1 Baseline patient characteristics

In this study, 304 patients were selected with AMI, including 76 patients with FWR and 228 patients with non‐CR. Among the 304 patients, the average age of the FWR group was 71.30 ± 9.58 years old, and that of the non‐CR group was 63.03 ± 12.65 years old, which was statistically different (*p *< 0.001). As shown in Table 1, the results of neutrophil percentage, MLR and NPAR were higher in the FWR group than in the non‐CR group (*p *< 0.05). In contrast, compared with the FWR group, SBP and albumin levels were significantly higher in the non‐CR group (*p *< 0.05). In addition, the proportion of emergency PCI patients in the non‐CR group was higher than that in the FWR group, and the difference was statistically significant (*p *< 0.001). No matter in the number of women, hypertension, diabetes mellitus, previous stroke, previous coronary artery disease (CAD), smoker, drinker, statin treatment, or the levels of DBP, heart rate between the two groups, there were no significant difference (all *p *> 0.05). Besides, criminal blood vessels, including left anterior descending (LAD), left circumflex artery (LCX), and right coronary artery (RCA), had no difference in both groups.

**TABLE 1:** Characteristics of the patients at baseline

Variables	non‐CR (*n* = 228)	FWR (*n* = 76)	*p*‐value
Age, years	63.03 ± 12.65	71.30 ± 9.58	<0.001
Women, *n* (%)	61 (26.8%)	28 (36.8%)	0.094
Hypertension, *n* (%)	123 (53.9%)	44 (57.9%)	0.549
Diabetes mellitus, *n* (%)	50 (21.9%)	17 (22.4%)	0.936
Previous stroke, *n* (%)	10 (4.4%)	6 (7.9%)	0.374
Previous CAD, *n* (%)	35 (15.4%)	7 (9.2%)	0.179
Smoker, *n* (%)	92 (40.4%)	37 (48.7%)	0.203
Drinker, *n* (%)	43 (18.9%)	14 (18.4%)	0.932
SBP, mmHg	129 ± 23.14	120.96 ± 26.59	0.012
DBP, mmHg	71.59 ± 11.20	71.12 ± 16.69	0.780
Heart rate, beat/min	80 (67, 91.75)	80 (70, 100)	0.222
Emergency PCI, *n* (%)	180 (78.9%)	28 (36.8%)	<0.001
Criminal vessel			0.068
LAD, *n* (%)	101 (49.5%)	19 (45.2%)	
LCX, *n* (%)	37 (18.1%)	14 (33.3%)	
RCA, *n* (%)	66 (32.4%)	9 (21.4%)	
Neutrophil percentage, %	77.30 ± 10.81	82.63 ± 7.07	<0.001
Albumin, g/L	39.23 ± 5.47	35.14 ± 4.45	<0.001
NPAR	2.02 ± 0.49	2.40 ± 0.38	<0.001
Monocyte count, ×10^9^/L	0.47 (0.32, 0.68)	0.77 (0.49, 0.96)	<0.001
Lymphocyte count, ×10^9^/L	1.41 (0.93, 1.86)	0.99 (0.70, 1.29)	0.001
MLR	0.34 (0.23, 0.52)	0.76 (0.45, 1.22)	<0.001
ACEI/ARB treatment, *n* (%)	101 (44.3%)	11 (14.5%)	<0.001
β‐blocker treatment, *n* (%)	122 (53.5%)	25 (32.9%)	0.002
Statin treatment, *n* (%)	224 (98.2%)	73 (96.1%)	0.508

Abbreviations: ACEI/ARB, angiotensin‐converting enzyme inhibitor/angiotensin receptor blocker; CAD, coronary artery disease; DBP, diastolic blood pressure; LAD, left anterior descending; LCX, left circumflex artery; MLR, monocyte‐to‐lymphocyte ratio; NPAR, neutrophil percentage to albumin ratio; PCI, percutaneous coronary intervention; RCA, right coronary artery; SBP, systolic blood pressure.

### 3.2 Results of logistic regression analysis

Age, SBP, emergency PCI, criminal vessel, NPAR, MLR, ACEI/ARB, and β‐blocker treatment were the risk factors of the FWR (all *p *< 0.05). Moreover, the results showed that emergency PCI (OR = 0.27, 95% CI: 0.094–0.751, *p* = 0.012), ACEI/ARB treatment (OR = 0.17, 95% CI: 0.044–0.659, *p* = 0.010), NPAR (OR = 2.69, 95% CI: 1.031–7.044, *p* = 0.043), and MLR (OR = 5.99, 95% CI: 2.09–17.168, *p* = 0.001) were the independent risk factors of the FWR patients with AMI (Table 2).

**TABLE 2:** The results of logistic regression analysis

Variables	Univariable analysis		Multivariable analysis	
	OR (95% CI)	*p*‐value	OR (95% CI)	*p*‐value
Age	1.06 (1.038–1.091)	<0.001	1.02 (0.978–1.068)	0.329
Women	1.60 (0.921–2.77)	0.096	–	–
SBP	0.99 (0.975–0.997)	0.013	0.98 (0.963–1.006)	0.160
Emergency PCI	0.17 (0.099–0.302)	<0.001	0.27 (0.094–0.751)	0.012
Criminal vessel		<0.001		0.275
LAD	0.13 (0.065–0.272)		0.74 (0.209–2.613)	
LCX	0.27 (0.119–0.599)		1.52 (0.369–6.263)	
RCA	0.10 (0.04–0.23)		0.33 (0.07–1.578)	
NPAR	5.23 (2.632–10.378)	<0.001	2.69 (1.031–7.044)	0.043
MLR	9.81 (4.117–23.354)	<0.001	5.99 (2.09–17.168)	0.001
ACEI/ARB treatment	0.21 (0.107–0.424)	<0.001	0.17 (0.044–0.659)	0.010
β‐blocker treatment	0.43 (0.247–0.734)	0.002	2.46 (0.873–6.945)	0.089

### 3.3 Results of ROC curve analysis

**FIGURE 1:**
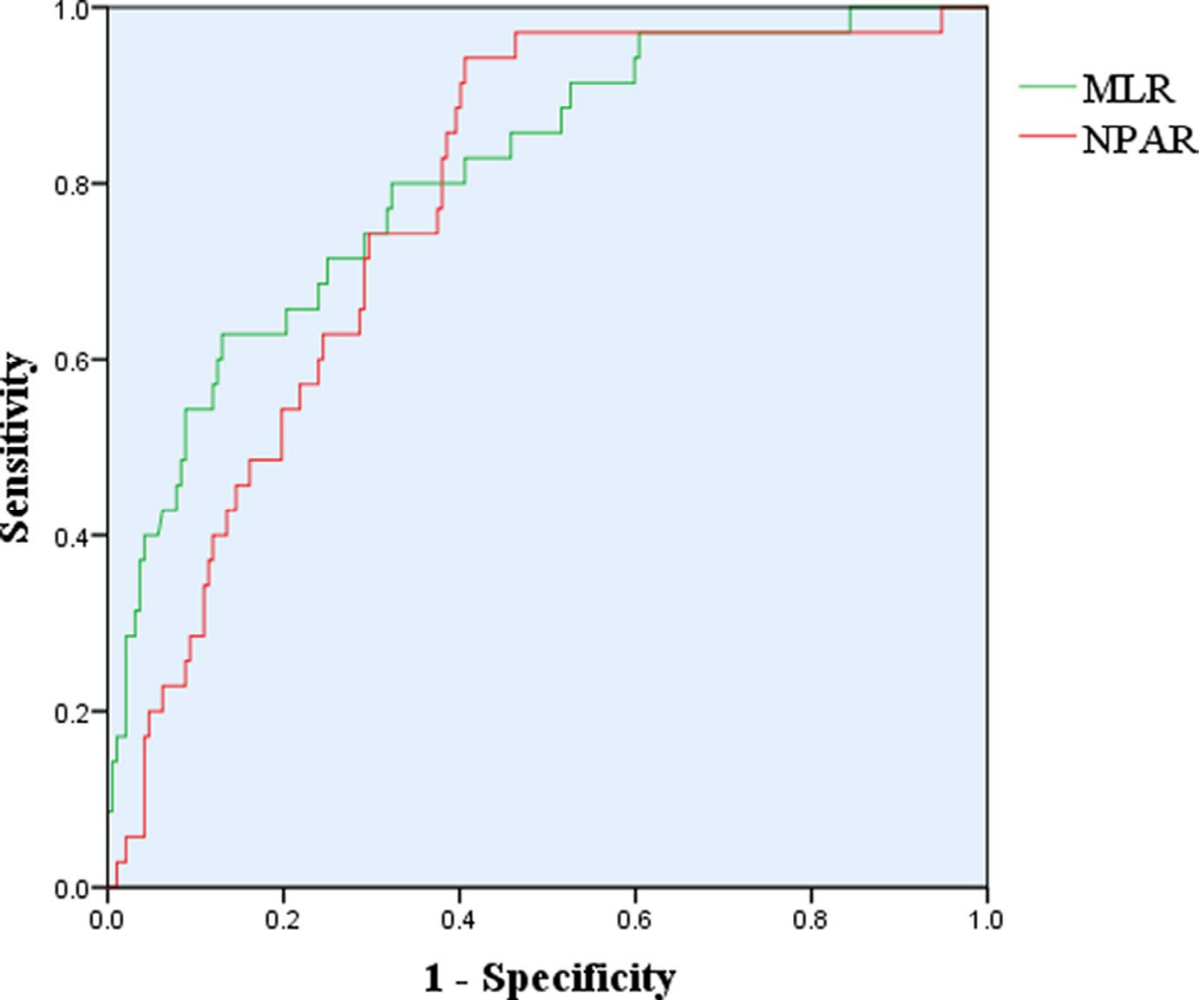
Receiver‐operating characteristic curve of NPAR and MLR for predicting FWR patients with AMI

The level of MLR at least 0.64 was predictive of FWR with 62.2% sensitivity and 86.4% specificity (AUC = 0.811, 95% CI: 0.734–0.889, *p*<0.001). Additionally, the meaningful result of NPAR was also obtained (AUC = 0.778, 95% CI: 0.705–0.850, *p *< 0.001) with 90.6% sensitivity and 60% specificity, and the cutoff was 2.02 (Table 3, Figure 1).

**TABLE 3:** The predictive value of MLR and NPAR on FWR after STEMI

Variables	AUC	Sensitivity (%)	Specificity (%)	95% CI	*p*‐value
MLR	0.811	62.2	86.4	0.734–0.889	<0.001
NPAR	0.778	90.6	60	0.705–0.850	<0.001

## 4 DISCUSSION

FWR is the deadliest type of CR, so it is particularly important to predict the occurrence of FWR as soon as possible through effective and sensitive indicators for its early prevention.^16^ In this study, emergency PCI, ACEI/ARB therapy, NPAR and MLR were influencing factors of the FWR patients with AMI independently. According to the ROC curve analysis, NPAR and MLR can better predict FWR, respectively.

Emergency PCI and ACEI/ARB treatment were independent protective factors for FWR patients. Emergency PCI within 12 hours after onset can recanalize the occluded coronary artery and perfuse the myocardium, reducing the area of myocardial necrosis and significantly improving the prognosis of patients.^17^ Thus, emergency PCI is considered to be an important measure to reduce the development of FWR.^18^ Early application of ACEI/ARB treatment can alleviate the process of ventricular remodeling after AMI and reduce the possibility of FWR.^4^

NPAR and MLR are new markers by integrating two indicators, respectively, which can provide more information than each alone. This study showed that the high level of NPAR and MLR were independent risk factors for FWR patients, with sensitive indicators for predicting FWR. NPAR, composed of neutrophil percentage and albumin, represents two different mechanisms that lead to FWR, and there may be a synergistic effect between them. After the occurrence of AMI, the ischemic myocardial cells are extensively necrotic, which leads to severe inflammatory reaction.^19^ Necrotic cardiomyocytes and matrix fragments activate complement amplification cascade reaction and Toll‐IL‐1 receptor pathway, and then nuclear factor (NF)‐κB is activated to induce the secretion of chemokines, cytokines, and adhesion molecules, which leads to neutrophil infiltration.^20^ Neutrophil's excessive production of reactive oxygen species and proteolytic enzymes increases the risk of FWR.^21^ The decrease of plasma albumin level may affect the ability of myocardial fiber regeneration and repair.^22^ Cui et al have shown that NPAR is independently related to hospital mortality in patients with STEMI.^12^ Wang et al have also found that with the increase of NPAR, the risk of all‐cause death in patients with acute kidney injury is higher.^23^ MLR, combining monocyte and lymphocyte, is closely related to adverse cardiovascular events in patients with coronary artery disease.^24^ Monocytes can migrate from blood to tissues in response to body signals and differentiate into inflammatory dendritic cells, macrophages, and foam cells, thus activate the secretion of proinflammatory cytokines, resulting in the destruction and dissolution of cardiomyocyte membrane structure and fibrin cytoskeleton.^25^ Previous studies have shown that lymphocyte count is negatively correlated with inflammatory response, and lower lymphocyte count increases the risk of cardiovascular events and mortality.^26^ In inflammatory state, lymphocytes are recruited into myocardial infarction, and the immune system will also produce a group of T cells, which can inhibit inflammatory reaction.^27^ Kurtul et al have shown that MLR is associated with adverse hospital outcomes in STEMI patients undergoing PCI.^28^ Fan et al also reported that high levels of MLR in AMI patients treated with PCI were independently related to the risk of 6‐month death.^29^ In the study of 306 patients, Wang found that MLR was an independent predictor of major adverse cardiovascular events in STEMI patients.^13^

This study took the lead in exploring the relationship between NPAR, MLR, and CR. NPAR and MLR, as easily obtained new inflammatory indicators, have certain predictive value for FWR patients and can quickly select high‐risk patients with FWR. Early intervention on these patients can improve their prognosis.

This study is a retrospective study with relatively few samples, and high‐quality randomized controlled clinical trials are needed to further verify the clinical significance of NPAR and MLR for FWR. In addition, the definition of FWR in this study was judged by visual assessment, which may bring bias to the diagnosis of FWR. It is expected that there will be more accurate and sensitive clinical indicators to predict the development of FWR in order to intervene as soon as possible.

## 5 CONCLUSION

Emergency PCI and ACEI/ARB treatment were independent protective factors for FWR patients with AMI, while the increase of MLR and NPAR were independent risk factors. What's more, NPAR and MLR are good indicators for predicting FWR. This study provides evidence for a better understanding between NPAR, MLR, and FWR.

## CONFLICT OF INTERESTS

The authors declared that there were no potential conflicts of interest with the research, authorship, and publication of this article.

## Data Availability

The data that support the findings of this study are available on request from the corresponding author. The data are not publicly available due to privacy or ethical restrictions.
